# Analysis of oral microbiome on temporary anchorage devices under different periodontal conditions

**DOI:** 10.1186/s40510-023-00488-x

**Published:** 2023-10-30

**Authors:** Ningrui Zhao, Qian Zhang, Yanning Guo, Shengjie Cui, Yajing Tian, Yanheng Zhou, Xuedong Wang

**Affiliations:** 1 Department of Orthodontics, Peking University School and Hospital of Stomatology, 22# Zhongguancun South Avenue, Beijing, 100081 China; 2 National Clinical Research Center for Oral Diseases & National Engineering Laboratory for Digital and Material Technology of Stomatology, Beijing, 100081 China; 3 Central Laboratory, Peking University School and Hospital of Stomatology, Haidian District, Beijing, China

**Keywords:** Temporary anchorage devices, Periodontitis, Microbiome, Orthodontic treatment

## Abstract

**Background:**

Temporary anchorage devices (TADs) are maximum anchorages that have been widely used in orthodontic treatment. The aim of the study was to uncover whether a history of periodontitis would influence microbiome colonization on the TAD surface.

**Results:**

Patients were grouped by periodontal evaluations before the orthodontic treatment. Patients with healthy periodontal conditions were classified as the healthy group, and patients diagnosed with periodontitis stage II or even worse were classified as the periodontitis group. Scanning electron microscopy (SEM) was used to analyze the existence of biofilm on the surface of 4 TADs from the healthy group and 4 TADs from the periodontitis group. Fifteen TADs from the healthy group and 12 TADs from the periodontitis group were collected. The microorganisms on the surface of TADs were harvested and analyzed by 16S rRNA gene sequencing. α-diversity indices and β-diversity indices were calculated. Wilcoxon’s test was used to determine differences between genera, species as well as KEGG functions. SEM analysis revealed bacteria colonization on the surface of TADs from both groups. Principal coordinate analysis (PCoA) based on β diversity revealed differential sample clusters depending on periodontal conditions (*P* < 0.01). When comparing specific genera, *Fusobacterium, Porphyromonas, Saccharibacteria_(TM7)_[G-1]*,* Dialister, Parvimonas, Fretibacterium, Treponema* were more enriched in TADs in the periodontitis group. In the KEGG analysis, TADs in the periodontitis group demonstrated enriched microbial activities involved with *translation, genetic information processing, metabolism,* and *cell motility*.

**Conclusions:**

This analysis elucidated the difference in total composition and function of TADs oral microorganisms between patients periodontally healthy and with periodontitis.

## Introduction

Anchorage control has been a major concern for orthodontists for decades. As maximum anchorage or skeletal anchorage, the temporary anchorage devices (TADs) have been well received since their appearance. This device facilitates anchorage reinforcement compared to conventional anchorage control and has great advantages in flexibility, versatility, minimal invasiveness, and independence of patient compliance [1, 2]. It is particularly indicated in anterior en masse retraction, molar protraction, as well as the intrusion of supra-erupted teeth, and midline correction [3]. However, on some occasions, TADs present excessive mobility which eventually leads to loss of anchorage. Though the failure rate of TADs is generally considered under 5–15% [4, 5], the rising demand for TADs in clinical use significantly gives rise to more individuals suffering from postoperation complications.

TADs are implanted in the alveolar bone between dental roots and are exposed to all sorts of oral microorganisms. Once inserted, an artificial sulcus is created, allowing for the invasion of the oral microbiome. It is probable that periodontal pathogens could penetrate the epithelial junction and induce inflammation around TADs, which might further lead to their failure [6, 7]. Several attempts have been made to the observation of biofilm attached to the surface of TADs. Ferreira et al. revealed biofilm existence on the head, transmucosal, and body segments of the TADs through scanning electron microscopy (SEM) [8]. With the utilization of fluorescence imaging, Garcez et al. observed higher fluorescent intensity in inflamed TADs with redness on surrounding tissues [9]. Although some studies attempted to explain the failure of TADs through the discovery of well-known periodontal pathogens, there is controversy in whether the oral microbiome plays a pivotal role [10–12]. Studies showing the full picture of the microbiome of TADs are still lacking.

Periodontitis is a multifactorial inflammatory disease that is initiated by subgingival dental biofilm and eventually causes the irreversible destruction of the periodontium [13]. MicroRNAs, transglutaminases, and circulating cells are all considered important modulators in the development of periodontitis [14–18]. Routine treatment of periodontitis includes root scaling and planing (RSP) and surgical intervention. Coadjuvant use of antibiotics and immune response modulators, for instance, cyclosporine A and tacrolimus, has been brought to light in the treatment sequence [19, 20].

As more and more patients with periodontitis are seeking orthodontic treatment today, there is an elevated use of TADs in orthodontic patients with a history of periodontitis. Subgingival periodontal pathogens might migrate into TAD insertion sites and cause inflammation of surrounding soft and hard tissue, as analogous to peri-implantitis [21, 22]. In this study, we hypothesize that there is a difference in the adhesion of pathogenic oral microorganisms around TADs from patients with healthy periodontal conditions or patients with a history of periodontitis.

This analysis takes the form of a pilot study of the microbiome on the surface of TADs between orthodontic patients with healthy periodontal conditions and with a history of periodontitis. We employed 16S rRNA gene sequencing to analyze the microbiome, as it could detect population diversity, identify the structure of the microbiome, and predict functional roles on certain occasions [23]. The aim of the study was to uncover whether a history of periodontitis would influence microbiome colonization on the TAD surface. This study highlights how periodontal conditions influence the microbiome community on the surface of TADs.

## Materials and methods

### Participant selection and sample collection

All of the subjects in this study participated in orthodontics treatments in Peking University Hospital of Stomatology. In all of them, the use of orthodontic anchorage was indicated. Each participant signed an informed consent form to enroll in the trial. This analysis was ratified by the Ethics Committee of the Peking University Hospital of Stomatology under PKUSSIRB-202060204**.** All methods were carried out following relevant guidelines and regulations.

In this analysis, periodontitis patients were classified in periodontitis stage II or even worse (interdental clinical attachment loss (CAL) at sites of greatest loss >  = 3 mm, radiographic bone loss >  = 15% of the root) according to the 2017 classification [24]. At their initial visits, their maximum probing depth was >  = 5 mm. After systematic periodontal treatment, a stable periodontal condition (no probing depth > 4 mm, plaque index < 30%, gingival index < 30%, and no occlusal trauma) was obtained from these patients before orthodontic treatment. In the healthy group, patients were presented with no periodontitis (probing depth < 3 mm, no CAL). Other inclusion criteria included: (1) aged 12–45 years; (2) non-smokers; (3) without systematic disease; (5) not pregnant; and (6) no antibiotics used up to three months before removal. In total, the periodontitis group included 12 well-controlled periodontitis patients undergoing orthodontic treatment for 16S rRNA analysis, and 4 patients for SEM analysis. The healthy group included 15 periodontally healthy patients under orthodontic treatment for 16S rRNA analysis and 4 patients for SEM analysis. The “Micropower” package (http://github.com/brendankelly/micropower) was used to assess the sample size.

Self-drilling titanium orthodontic TADs (diameter, 1.5 mm; length, 7 mm or 8 mm; Zhongbang Medical Treatment Appliance, Xi’an, China) were inserted in the maxilla, between tooth roots of anterior or posterior teeth, between the buccal or palatal surface. To be specific, the insertion sites were between maxillary lateral incisor and canines, between the first premolar and second premolar, and between the second premolar and the first molar. All TADs were inserted by one experienced orthodontist. No damage to the adjacent tooth roots was observed. All patients received oral hygiene instructions to brush TADs and the surrounding tissues when adopting oral hygiene methods. All TADs were activated 1 month after placement. In total, we observed 8 TADs under SEM, 4 from periodontitis, and 4 from the healthy group. Twenty-seven TADs from 27 patients, 12 from the periodontitis group, and 15 from the healthy group were included for 16S rRNA gene sequencing. All of the included TADs remained stable during treatment and were removed until attaining the desired result.

### SEM analysis

Eight TADs from 8 individuals were observed under SEM to scrutinize the biofilm attached. After removal, the TADs were transferred to 1.5 mL nonpyrogenic microcentrifuge tubes containing 0.5 mL 4% glutaraldehyde and stored at − 4 °C. Before observation, the TADs were dehydrated in increasing concentrations of ethanol (30%, 50%, 70%, 80%, 90%, 95%, and 100%). After conductive coating, they were sent for examination under SEM (SU8010, Hitachi, Tokyo, Japan).

### DNA extraction

The TADs were placed in nonpyrogenic microcentrifuge tubes containing 0.5 mL normal saline solution and stored at − 20 °C refrigerator temporarily. Before DNA extraction, the tubes were agitated in an ultrasound cleaner (SB-3200DTN, Scientz, Ningbo, China) for 20 min. The tubes were then centrifuged at 8000 rpm for 15 min to remove the supernatant. The precipitate was then sent for DNA extraction.

QIAamp DNA Mini Kit (QIAGEN, Hilden, Germany) was used to extract genome DNA. Extraction procedures were performed according to the kit instructions. Before extraction, 180 μL lysozyme (Solarbio, Beijing, China) was added to the reaction system. The system was then incubated at 37 °C for 30 min. NanoDrop 2000 Spectrophotometer (Thermo Fisher Scientific, Carlsbad, CA, United States) and 1% agarose gel electrophoresis were used to determine the purity and integrity of DNA.

### 16S rRNA gene sequencing

The V3-V4 region of the bacteria 16S ribosomal RNA genes was amplified by Polymerase chain reaction (PCR) (95 °C for 3 min, followed by 30 cycles at 98 °C for 20 s, 58 °C for 15 s, and 72 °C for 20 s and a final extension at 72 °C for 5 min) using barcoded primers 341F 5′-CCTACGGGRSGCAGCAG-3′ and 806R 5′-GGACTACVVGGGTATCTAATC-3′. PCR reactions were performed in a 30 μL mixture containing 15 μL of 2 × KAPA Library Amplification ReadyMix, 1 μL of each primer (10 μM), 50 ng of template DNA, and ddH2O. Negative controls consisting of empty sterile storage tubes were processed for DNA extraction, and amplification using the same procedures and reagents used for the TAD samples. There was no detectable amplification in the negative controls. Amplicons were extracted from 2% agarose gels and purified using the AxyPrep DNA Gel Extraction Kit (Axygen Biosciences, Union City, CA, U.S.) according to the manufacturer’s instructions and quantified using Qubit®2.0 (Invitrogen, U.S.). All quantified amplicons were pooled to equalize concentrations for sequencing using Illumina MiSeq (Illumina, Inc., CA, USA). The paired-end reads of 250 bp were overlapped on their 3 ends for concatenation into original longer tags by using PANDAseq (https://github.com/neufeld/pandaseq, version 2.9). DNA extraction, Library construction, and sequencing were conducted at Realbio Genomics Institute (Shanghai, China).

### Data processing

Raw data were deposited at Sequence Read Archive under project No.PRJNA910988. Preprocessing of data was performed under the guidance [24]. After demultiplexing, Vsearch [25] (version 2.15) was used to merge raw paired-end sequences according to the overlap of the paired-end reads, allowing for a maximum of five mismatches. Barcode and primers were then removed allowing a maximum error rate of 1% by Vsearch [25] (version 2.15) to obtain clean reads. After dereplication, unoise3 in USEARCH [26] was used to denoise to amplicon sequence variance (ASV) for representative sequence selection. Next, Vsearch [25] (version 2.15) was utilized to detect and exclude chimeras. The feature table was created by using Vsearch [25] (version 2.15). RDP classifier and the Human Oral Microbiome Database [27, 28] were both employed as databases in sequence annotation.

To start the downstream analysis, random rarefication procedures were taken for each pre-processing sequence to mitigate the effect of varying sequencing depths. α-diversity indices (the Chao1 richness estimator, Shannon index) were calculated as metrics for the microbial diversity within each sample. Bray Curtis distance and Unifrac distance were assessed as representations of the overall microbiome dissimilarities or β-diversity. Principal coordinate analysis (PCoA) was implemented to reflect the β-diversity through R. Then, ASVs were classified into microbial taxa (phylum, class, order, family, and genus). The phylogenetic tree was constructed on ITOL (https://itol.embl.de/). Linear discriminant analysis Effect Size (*LEfSe)* was used to identify differential taxa between groups [29]. Phylogenetic Investigation of Communities by Reconstruction of Unobserved States (PICRUSt) [30] (version 1.1.3) tool was adopted to predict functional roles based on the Kyoto Encyclopedia of Gene and Genomes (KEGG) pathway. We have conducted the MaAsLin (Multivariate Analysis by Linear Models) (http://huttenhower.sph.harvard.edu/galaxy/) to address potential biases.

### Statistical analysis

An independent sample Student’s *t* test and nonparametric Wilcoxon’s test were used to evaluate demographic features and clinical parameters between the two groups. The difference between α- diversity was calculated by analysis of variance (ANOVA). The pairwise permutational multivariate analysis of variance (PERMANOVA) procedure tested the significance of β-diversity between groups. This was realized by the Adonis function of the R package vegan 2.5–6, allowing for 9999 permutations. Wilcoxon’s test was used to determine differences between genera, species as well as KEGG functions. False detection rate (FDR) correction was employed. Data were plotted using the GraphPad Prism program (GraphPad Prism 9.0).

## Results

### Overview of subjects and samples

In this study, we performed SEM observation on 8 samples from 8 individuals, 4 from the periodontitis group and 4 from the healthy group. Then, we conducted 16S rRNA gene sequencing of 27 TADs samples from 27 individuals, 12 from the periodontitis group and 15 from the healthy group. The individuals’ demographic features and clinical parameters are presented in Table 1 for the 16S rRNA sequencing analysis. No detection between microbial measurements and clinical metadata (age, sex, TAD insertion days, Angle’s classification) was found in the MaAsLin analysis.

**Table 1:** Demographic features of patients under 16S rRNA gene sequencing

Variable	Periodontitis (n = 12)	Healthy (n = 15)	*P*
Age (y)	30.33 ± 9.36	22.4 ± 7.00	0.02
Sex ratio			0.075
Male	3	0	
Female	9	15	
Time in oral cavity (days)	774.83 ± 229.15	622.33 ± 299.25	0.159
Angle’s classification			0.407
I	4	3	
II	4	9	
III	4	3	

### SEM proving the existence of bacteria

**Fig. 1:**
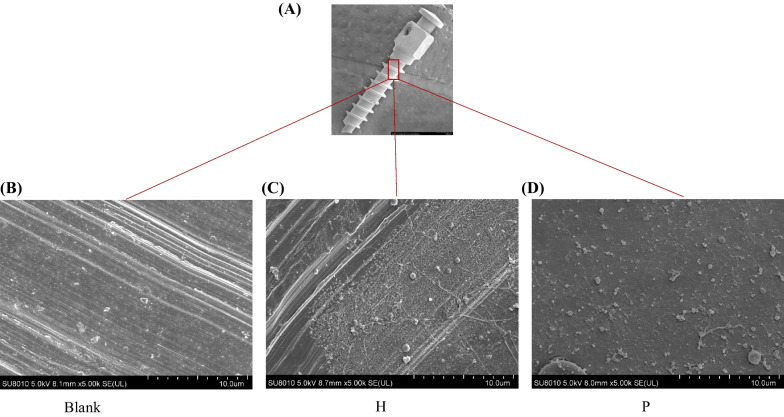
SEM images of TADs showing microbiome colonization on its surface. **A** The whole picture of the TAD. **B** Higher magnification of new sterilized TAD × 5.00 k. **C** Higher magnification of the transmucosal segment of TAD with tissues and biofilm formation × 5.00 k of the healthy group. **D** Higher magnification of transmucosal segment of TAD with bacteria × 5.00 k of the periodontitis group

To explore the microbiome on the TADs’ surface, we first performed SEM to prove the existence of the microbiome on TADs (Fig. 1). SEM demonstrated the existence of microflora on the surface of observed TADs both in the periodontitis group and in the healthy group. Rods and coccoid bacteria were all seen in this region. Besides, tissue remnants containing fibers and red blood cells were also observed. This testified to microbiome colonization on the surface of TADs.

### Phylogenetic alterations under different periodontal conditions

During 16S rRNA gene sequencing processing, a total of 1,232,565 clean reads were acquired. The average sequences for each sample were 45,650, eliciting 1591 ASVs. After rarefication, each sample contained 25,975 clean reads.

**Fig. 2:**
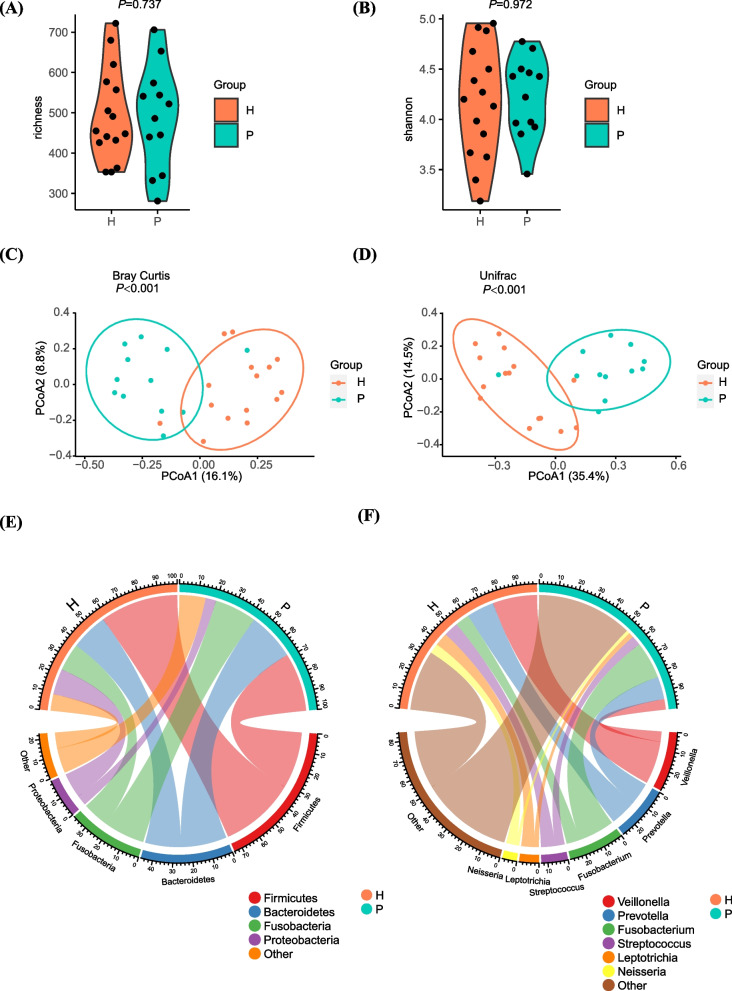
Comparisons between P and H microbial communities. **A** A boxplot of α-diversity richness index between groups (*P* = 0.737). **B** A boxplot of α-diversity Shannon index between groups (*P* = 0.972). **C** Principal coordinate analysis (PCoA) based on Bray Curtis distances is shown for the periodontitis group (green) and healthy group (pink) (p < 0.001). **D** Principal coordinate analysis (PCoA) based on Unifrac distances (*P* < 0.001). **E** Plot of the most abundant phylum of P and H group. **F** Plot of the most abundant genera of P and H group

To characterize the microbiome of the individuals with chronic periodontitis, α-diversity and β-diversity were first evaluated as reflections of the overall structural features and composition (Fig. 2). No statistical difference was observed in α-diversity indices between the periodontitis group and healthy group (*P* = 0.737, *P* = 0.972, respectively) (Fig. 2A, B). However, PCoA based on weighted Bray Curtis distances revealed a statistically significant discrepancy in phylogenetic structures between the periodontitis group and the healthy group (*P* < 0.001) (Fig. 2C). Different clusters were formed, indicating separation in microbiome composition between the groups. Similarly, PCoA based on Unifrac distances revealed a clear separation between the groups (*P* < 0.001) (Fig. 2D).

### Identification of microbiota composition

Next, we classified ASVs into certain microbial taxa to identify the compositional changes in individuals under different periodontal conditions. In general, we discovered 12 phyla, 25 classes, 38 orders, 62 families, and 113 genera. Phylum, such as *Firmicutes, Bacteroidetes*, *Fusobacteria*, and *Proteobacteria,* constituted the majority of the microbiota on the TADs (Fig. 2E). Genera including *Veillonella, Prevotella, Fusobacterium, Streptococcus, Leptotrichia, and Neisseria* predominated (Fig. 2F).

**Fig. 3:**
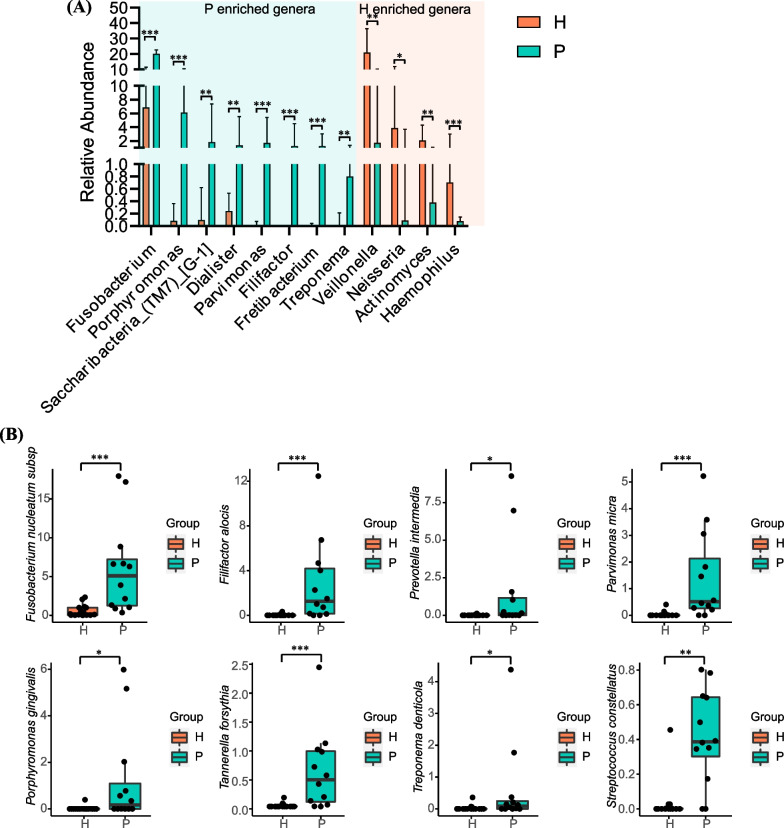
Differential genera between P and H based on Wilcoxon test. **A** The bar plot depicts the differential genera between P and H (*P* < 0.05). **B** The box plot depicts the differential species between P and H (*P* < 0.05)

To better characterize the differences, Wilcoxon’s test was used. The bar plot showed the differential microbiome between the periodontitis group and the healthy group (Fig. 3A). At the genus level, *Fusobacterium*, *Porphyromonas, Saccharibacteria_(TM7)_[G-1], Dialister, Parvimonas, Fretibacterium, Treponema* were more enriched in the periodontitis group (*P* < 0.05). *Veillonella, Neisseria, Actinomyces, Haemophilus* were more enriched in the healthy group (*P* < 0.05). At the species level, we identified differential species associated with periodontal disease. *Fusobacterium nucleatum*, *Filifactor alocis*, *Prevotella intermedia*, *Parvimonas micra*, *Porphyromonas gingivalis*, *Tannerella forsythia*, *Treponema denticola*, and *Streptococcus constellatus* demonstrated significant higher relative abundance in periodontitis group compared with healthy group (Fig. 3B).

### Microbiota involvement in functional variation

**Fig. 4:**
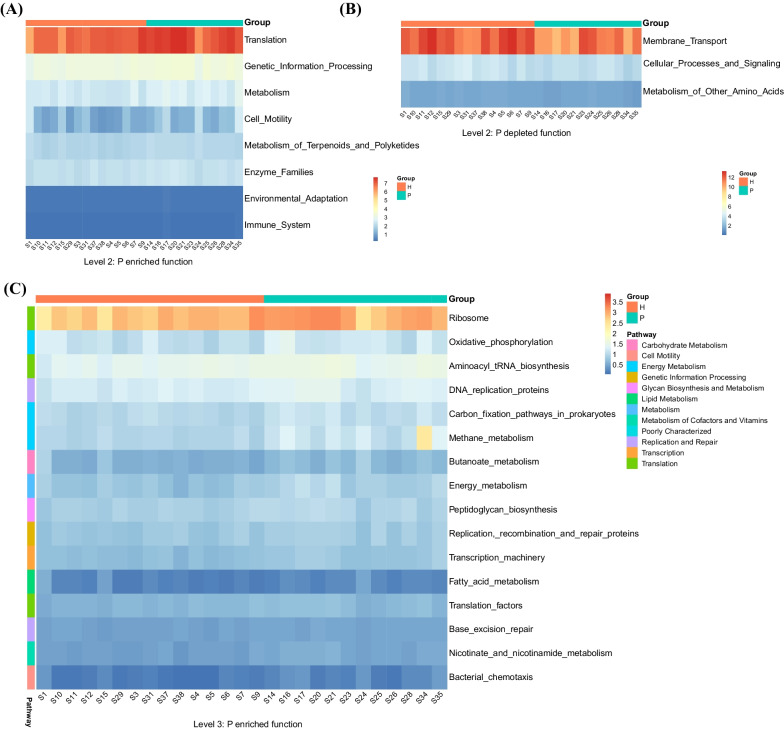
Differential functions based on KEGG database and Wilcoxon test. **A** Enriched functional pathways of periodontitis group on Level 2. PICRUSt tool was adopted to predict functional roles based on KEGG database. Differentially abundant functional pathways were listed in rows and columns. **B** Enriched functional pathways of the healthy group on Level 2. **C** Enriched functional pathways of periodontitis group on Level 3

To study the functional changes in TADs in individuals with different periodontal conditions, the PICRUSt algorithm was employed to predict the path of microbiota derivation based on the KEGG database. Differences in functional abundance between TADs in patients with periodontitis and TADs in patients in good periodontal health were evaluated (Fig. 4). The periodontitis group demonstrated enriched microbial activities involved with *translation, genetic information processing, metabolism,* and *cell motility* on KEGG Level 2 (Fig. 4A). To be more specific, on KEGG Level3, enriched functions in periodontitis group were observed in *Ribosome*, *Oxidative phosphorylation, Aminoacyl tRNA biosynthesis, DNA replication proteins, *etc. (Fig. 4C)*.* TADs on healthy periodontal individuals demonstrated enriched functions in *Membrane Transport, Cellular Processes and Signaling, Metabolism of Other Amino Acids* (Fig. 4B).

## Discussion

Our study characterized alterations in microbial community profiles on the TADs surface depending on the periodontal condition. SEM analysis demonstrated the microbiome colonization on the surface of observed TADs both in the periodontitis group and in the healthy group. We identified the compositional and phylogenetic changes in the microbiome on the surface of TADs in relation to their periodontal condition. We also predicted the functional involvement of the microbiome on patients with periodontitis. To the best of our knowledge, this is pioneering research in elucidating the influence of periodontitis history on the microbiome colonization on the surface of TADs.

The microbiome normally colonizes on the surface of TADs. When a TAD is inserted, a new site is created, which is defined as the gingival sulcus between the surrounding gingiva and the TAD cervical [31]. In a previous study utilizing SEM, Ferreira discovered bacteria colonization on the head, transmucosal surface, and body segment of TADs [8]. Similarly, in our study, the existence of microflora was observed on the surface of TADs. Previous studies also observed the adhesion, aggregation, and development of the microbial colonization process in TADs using cell growth methods or fluorescence images [9, 31]. The interactions between the microorganisms and the host maintain the microecological balance around the TADs [10].

Periodontitis is not a contraindication for orthodontic treatment. Except for measurement of the clinical attachment level, probing depth, and radiographic assessment of alveolar bone loss, early diagnosis of periodontitis might be realized through biomarkers, for instance, microRNAs and peptides [16, 32]. Traditional routine treatment of periodontitis includes RSP and surgical intervention. Recently, much attention has been drawn to immunomodulation. Drugs, stem cells, and other therapies targeting the immune microenvironment have shown great potential [33]. It has been confirmed that in periodontitis patients, orthodontic treatment would not bring additional damage to the periodontal tissue under patients’ strict plaque control methods [34]. In a meta-analysis, *Guo *et al. reported temporary increases in periodontal pathogens in orthodontic patients at the placement of the orthodontic appliance [35]. This disturbance would diminish several months later. According to later research of *Guo *et al., this phenomenon could also be observed in periodontitis patients receiving orthodontic treatment [36]. However, no previous studies have focused on microbiological evaluations of TADs, and the potential influence on patients’ periodontal condition.

In our study, we have discovered periodontal pathogens colonization on the TADs from the periodontitis group. At the species level, we discovered an increase in *Porphyromonas gingivalis, Tannerella forsythia, and Treponema denticola*, which were the component of the red complex in the Socransky’s analysis, in the periodontitis group [37]. An elevation of the orange complex components, *Fusobacterium nucleatum*, *Prevotella intermedia*, and *Streptococcus constellatus*, was also observed. We speculated that these pathogens might migrate from periodontal pockets to TAD surrounding tissue. The virulence factors of these microorganisms and their production of toxic metabolites might trigger the host inflammatory response, including the release of cytokines, and chemokines as well as the emigration of inflammatory cells [6]. Another research has already demonstrated oral microbiome dysbiosis contribute to the failure of TADs [38]. The result of our study reminds orthodontists to be fully aware of potential risks when applying TADs to periodontitis patients seeking orthodontic treatments.

Characterizing microbiome function is necessary to broaden our knowledge of how periodontal conditions affects the microbiome on the TADs’ surface. We used PICRUSt as a substitution method to characterize functional changes, which has been implemented in other sequencing studies [39–41]. Microbial activities involved with translation, genetic information processing, metabolism, and cell motility were abundant in the biofilm on TADs of the periodontitis group, which indicates functional dysbiosis. Demonstrating the role of the key bacteria that encode these functions and setting up the link between these functions and the mobility of TADs will be crucial in future research.

Our study discussed the relationship between the periodontal condition and microbiomes on the TADs surface using next-generation sequencing. Meanwhile, our study had a few limitations. The TADs samples were difficult to acquire. Each orthodontist would normally only insert a few TADs each month, and the sample size was relatively limited. Besides, each individual also exhibited individual variance in the oral microbiome composition. In addition, the age in the periodontitis group was a little higher than the healthy group. This could be explained by the natural progression of periodontitis often occurred with an increase in age. We have conducted the MaAsLin analysis to address the potential bias. Lastly, the golden standard for harvesting plaque samples is through a paper point or curette. Due to the size of the TAD, it was not feasible to employ the golden standard, so we employed the methods put forward by Andrucio [11]. Considering that periodontal condition affects TADs surrounding the microenvironment and could potentially influence the success of TADs, further research is needed to explain the mechanism of the oral microbiome and its relations with immobility.

## Conclusions

This analysis elucidated the difference in total composition and function of TADs oral microorganisms between patients periodontally healthy and with periodontitis. Periodontal pathogens, *Fusobacterium, Porphyromonas, Saccharibacteria_(TM7)_[G-1], Dialister, Parvimonas, Fretibacterium, Treponema* were more enriched in TADs from patients with a history of periodontitis.

## Data Availability

The datasets generated during and/or analyzed during the current study are available in the Sequence Read Archive repository (https://dataview.ncbi.nlm.nih.gov/object/PRJNA910988) under project No. PRJNA910988.

## Abbreviations

TADs: Temporary anchorage devices
SEM: Scanning electron microscopy
CAL: Clinical attachment loss
RSP: Root scaling and planing
PCoA: Principal coordinate analysis
PCR: Polymerase chain reaction
ASV: Amplicon sequence variance
*LEfSe*: Linear discriminant analysis effect size
PICRUSt: Phylogenetic Investigation of Communities by Reconstruction of Unobserved States
FDR: False detection rate
